# Trends in Distance Between Non-resident Parents and Minor Children Following Separation: Analysis of the Belgian Case, 1992–2018

**DOI:** 10.1007/s10680-023-09674-3

**Published:** 2023-08-08

**Authors:** Zuzana Zilincikova, Christine Schnor

**Affiliations:** 1 University of Groningen, Groningen, Netherlands; 2 UCLouvain, Louvain-la-Neuve, Belgium

**Keywords:** Separation, Divorce, Post-separation family, Non-resident parent, Geographical distance, Belgium, Register data

## Abstract

Geographic distance between a child and their non-resident parent is a key aspect of the reorganization of the family following parental separation. The increasingly equal involvement of both parents in the upbringing of their children is expected to translate into increasing geographic proximity between children and non-resident parents. So far, there has been no evidence about the time trends in geographical distances between minor children and non-resident parents outside of the Swedish context. In this study, we investigate these trends across Belgian separation cohorts from 1992 to 2018 and the extent to which they differ according to parental socioeconomic status and child’s age at separation. Overall, we observed a very small decrease in distance between children and their non-resident fathers and a somewhat larger decrease for non-resident mothers. The distance increased for very young children (0–2 years) and children with low-educated fathers. These findings point to inequalities in certain parent–child dyads.

## Introduction

In recent decades, fundamental changes have taken place in family life in most western societies. Increasing partnership instability has translated into an increasing number of post-separation families, and the organization of these families has changed. Whereas during the 1990s, separated fathers often had a limited role in the upbringing of their children (Vanassche et al., 2017), in recent years, more equal involvement of both separated parents has become more common (Westphal et al., 2014). In a number of European countries, recent family policies emphasized the rights and opportunities of separated fathers, thereby reinforcing current trends (Bernardi & Mortelmans, 2021). These trends may have important implications for the geographical proximity of children to their separated parents. In this study, we investigate trends in geographic proximity between minor children and non-resident parents across Belgian separation cohorts from 1992 to 2018 and the extent to which these differ according to parental socioeconomic status and child’s age at separation.

Geographic distance between non-resident parents and their children is a key aspect of the reorganization of the family following parental break-up. Geographic proximity plays an important aspect in maintaining the parent–child relationship (Viry, 2014), enables frequent face-to-face contact between parent and child (Mulder & van der Meer, 2009), and is usually a necessary condition for more equal involvement of both parents in childrearing (Bakker & Mulder, 2013). However, not all non-resident parents settle geographically close to their children. Geographic proximity between non-resident parent and a child is influenced by a number of factors, such as housing and employment opportunities, repartnering, and location of other family members. Additionally, geographic proximity may be limited by the resources of the parent as living near non-resident children may be financially demanding and thus not an option available to all. Second, not all parents may be motivated to continue to live near their children. Motivation for remaining nearby can be lower if the post-separation involvement of the non-resident parent in childrearing is limited to, for example, weekend visitations, as is often the case when children are young (Notaire.be, n.d.).

Existing empirical evidence reveals that the distance between a non-resident parent and a child tends to be relatively small. In Norway, 62% of non-resident separated parents lived within a 10 km radius of their child in 2012 (median 3.5 km for non-resident mothers and 5.4 km for non-resident fathers) (Dommermuth, 2016). In Sweden, about half of the non-resident parents separated in 2011 lived within 2 km of their children in the year after the separation (average distance 19 km for mothers and 25 km for fathers) (Turunen et al., 2023). Following the trends in distances over separation cohorts, Turunen et al. (2023) document for Sweden that the distance between child and non-resident parent decreased overall from the 1970s to the early 1990s and then stagnated from the mid-1990s until the end of their observation period in 2011. They also document that from the 1990s distances decreased for mothers and fathers in the highest-income group but remained the same or even increased for lower-income groups.

Until now, there has been no analysis of time trends in geographical distances between children and non-resident parents outside of the Swedish context. As Sweden deviates from other European countries in several areas, including societal value orientation and demographic behaviour (Ohlsson-Wijk et al., 2020), it remains unclear whether its trends may be indicative of those of other European countries. Increasing distance between children and separated parents with lower socioeconomic status could point to an increasing disadvantage for such children. There is also a lack of evidence on other key characteristics of non-resident parent–child pairs that are associated with the change in the geographic proximity across the cohorts. It is crucial to consider the age of the child, as we know that young children are more likely to live farther away from their non-resident parent (Dommermuth, 2016) and to lose the contact with them (Kalmijn, 2015). It thus seems that children who experience parental separation early in life are particularly disadvantaged: they live fewer years of their childhood with both parents than other children and have less contact with their non-resident parent after separation. Exploring the trends over time helps us to understand if the inequalities between non-resident parents and children with different characteristics (i.e. socioeconomic background and age) have widened or, on the other hand, have narrowed.

Belgium provides an interesting case study, as post-separation care arrangements there underwent major change in the past three decades. Empirical evidence from the northern, Dutch-speaking part of Belgium demonstrates that the proportion of divorced parents opting for shared physical custody increased from 8.5% in 1990–1995 to 36.7% in 2006–2008 (Vanassche, 2013), and the link between shared physical custody after separation and parental socioeconomic background became less pronounced (Schnor et al., 2017; Sodermans et al., 2013).

This paper enhances the understanding of the changing organization of post-separation families. We address (1) how geographical distance between child and non-resident parent one year after separation evolves across separation cohorts for 1992–2018 and (2) how these trends for separation cohorts differ for child–non-resident-parent pairs depending on social class (approximated by parental education) and the age of the child at separation. Understanding for which children the distances decrease, stagnate, or increase over separation cohorts reveals the extent of inequality and its trends among children experiencing parental separation. We also address whether the geographical proximity between child and non-resident parent changes in response to policies introduced in the observed period. The analysis is based on Belgian register–based data from 1992 to 2018.

## Parent–Child Ties After Separation

Many scholars link recent increases in separation and divorce rates to value change in society, characterized mainly by increased individualism. Unmarried cohabitation and easier access to divorce mean that the links between partners are in theory easily dissolved. However, the end of a union does not necessarily mean that ex-partners make decisions as single individuals but rather that their decisions are shaped by restructured family dynamics (Cooke et al., 2016). In practice, the lives of the ex-partners often remain linked when they have joint children, and their lives continue to be mutually affected (Settersten, 2015). For example, if a separated parent aims to move in together with a new partner, the location of the current partner and the ex-partner may need to be considered in the migratory decision, as the link through the child remains. Thus, the lives of ex-partners remain *linked* through their children, and their mobility decisions stay interconnected.

The proximity of separated parents seems to increase in importance with increased involvement of both parents in the upbringing of shared children (Amato et al., 2009; Dotti Sani & Treas, 2016; Henz, 2017; Sullivan et al., 2014; Westphal et al., 2014). In many western countries, the gender revolution and the increased involvement of women in the labour force resulted in men increasingly spending time caring for their children (Westphal et al., 2014). Consequently, fathers who were more involved prior to the separation also stay more involved in childcare after separation (Westphal et al., 2014). Daytime contact and overnight stays with fathers have increased over time (Westphal et al., 2014), and shared physical custody has become more popular (Vanassche et al., 2017). In parallel, the number of non-resident mothers (mothers registered at a different address than their child) has been also increasing. In the Swedish context, the proportion of non-resident mothers among parents separated in the previous year rose from 12 percent in 1974 to 26 percent in 2011 (Turunen et al., 2023). The legal system plays an important role in strengthening the role of both parents in post-divorce involvement with their children.

There is a complex relationship between geographical proximity and parental involvement and custody arrangements. Parental involvement and shared physical custody are predictors of geographical proximity between a non-resident parent and a child (Thomas et al., 2018; Viry, 2014). Although telecommunication can—at least to some extent—facilitate contact and co-parenting, it cannot replace more instrumental elements of parental care. Living in proximity is essential if care tasks are distributed more equally and both parents are involved in day-to-day care, such as dropping off or picking up children from school, preparing meals, etc. Geographical proximity is also one of the main aspects taken into consideration when establishing custody arrangements in court (Merla, 2021; Notaire.be, n.d.), and, conversely, shared physical custody can also limit the mobility of the parents involved. Still, it must be noted that even if parents live near one another they do not necessarily share childcare equally. Parental involvement is a result of the preferences of the non-resident parent but also the wishes and preferences of the children and the residential parent, and potentially court decisions (Dunn, 2004).

The patterns of involvement of non-resident fathers and mothers seem to differ. Research shows that non-resident mothers are more involved in instrumental support and in day-to-day aspects of children’s lives than non-resident fathers, whereas the former are less likely to provide financial support (Hawkins et al., 2006; Stewart, 2010). The quality of contact was also found to be higher for non-resident mothers than for non-resident fathers (Hawkins et al., 2006). In line with this, the average distance between a non-resident parent and a child is smaller if the non-resident parent is a mother (Dommermuth, 2016; Stjernström & Strömgren, 2012). In Sweden, non-resident mothers lived on average farther away than non-resident fathers during the 1970s, whereas in the late 2000s it was the reverse (Turunen et al., 2023).

The geographical distance between a child and its non-resident parent is a result of the migratory decisions of the separated parents. Migratory behaviour in separating parents is well-documented in the literature. Separated parents are more likely to move than parents in intact families (van der Wiel et al., 2021). Compared to childless ex-partners, ex-partners with children are less likely to migrate (Cooke et al., 2016; Spring et al., 2021; Thomas et al., 2017a, b), and if the ex-partners with children move, then the distance of a move tends to be shorter (Feijten & van Ham, 2013; Gram-Hanssen & Bech-Danielsen, 2008; Mulder & Malmberg, 2011; Thomas et al., 2017a, b). The sparse literature on time trends in the distance between a child and its non-resident parent is limited to Swedish context. These suggest that this distance changed over separation cohorts. Stjernström and Strömgren (2012) found for cross-sectional Swedish register data from 1990, 1995, 2000, and 2005 that in more recent years, non-resident parents tend to live in closer proximity to their children. Turunen et al. (2023) extended the previous research by studying geographical distances between non-resident parents and their children in the year immediately following the parental break-up, for separation cohorts 1974 to 2011. Their results showed a gradual decrease in distances between children and non-resident parents from the 1970s to the early 1990s, after which the trend stalled at a low level (approximately 25 km) for fathers while further slightly decreasing for mothers, especially in late 2000s (to 19 km). Furthermore, Turunen et al. (2023) do not find a direct effect of family policies on child–non-resident-parent distance. It remains unclear whether these trends are specific to the Swedish national context or whether similar findings would be observed in different national contexts.

Belgian family law has undergone several changes since the 1990s which are explained in detail in Sect. 5. The changes were in general aimed at more equal involvement of both parents in pre-separation and post-separation families and included reforms of parental leave and divorce law. Law changes are likely to reflect gradual societal change, and even though they may strengthen the ongoing societal trends, they might not have a shock effect on behaviour.

To sum up, the last three decades saw changes occur in the behaviour of parents, with the increasingly equal involvement of both parents in childrearing prior to as well as after the separation. These changes were reflected and potentially strengthened by changes in the law. Given these developments, *we hypothesize that the distance between separated parents decreased across 1992–2018 separation cohorts (H1).*

## The Privilege of Higher Social Classes?: Parental Education and Parent–Child Geographic Proximity

The link between parental socioeconomic status (approximated here by parental education^1^) and geographical proximity to children after separation is not clear-cut. On the one hand, higher-educated individuals are more mobile and move longer distances than their lower-educated counterparts (Feijten & van Ham, 2007). Thus, theoretically, we could expect highly educated parents to also be more mobile after partnership dissolution. In addition, highly educated separated individuals are more likely to repartner (de Graaf & Kalmijn, 2003), which tends to further increase the distance between ex-couple households (Stjernström & Strömgren, 2012; Thomas et al., 2017a, b). We could thus expect that higher parental education is associated with greater distances between non-resident parent and child. On the other hand, higher-educated fathers are more involved in the upbringing of their children; for example, higher-educated non-resident fathers have more overnight stays with their children than lower-educated (Westphal et al., 2014). Higher education is also found to be positively associated with shared physical custody (Juby et al., 2005; Sodermans et al., 2013). Finally, the desire to live near one’s children might be easier to realize for higher-educated parents who tend to have more resources that are often needed for securing suitable housing in closer proximity to their children (Turunen et al., 2023).

Previous research offers mixed results in terms of the relationship between parental education and the distance between non-resident parents and their children. A Swedish study using population register data has found that higher education is associated with shorter distance between parents and their non-resident children (Stjernström & Strömgren, 2012 (using data from 2005)). Viry (2014) using a Swiss sample and Thomas et al., (2017a, b) using a British sample of separated parents found no association between education and the distance between parental households. However, for the British context as well, Thomas et al. (2018) found that if both parents had at least a bachelor’s degree then the distances between them were greater. While no study focuses on geographical distance between separated parents in Belgium, Schnor and Mikolai (2020) documented that after separation Belgian mothers from disadvantaged backgrounds move farther and more often.

To the best of our knowledge, only one Swedish study has investigated how trends in distance between non-resident parents and children evolve within different social strata (even though they focus on income rather than educational attainment). Turunen et al. (2023) show that fathers in the highest-income quintiles lived farthest from their non-resident children during the 1970s and most of the 1980s and nearest during the 2000s. For non-resident mothers, the picture is less straightforward. High-earning non-resident mothers did not live farther away than mothers in the lowest-income groups throughout the cohorts of the 1970s to 1990s. However, high-earning mothers reduced the distance across the separation cohorts the most and were living by far nearest to their children throughout the 2000s. Similar evidence for the Belgian context is lacking. From the research on shared physical custody, which could point also to the trends in geographic proximity, we know that shared physical custody was initially mostly chosen by better-educated parents (Sodermans et al., 2013). This was likely because of the resources needed to maintain two homes and the level of post-divorce cooperation between the parents. Since 2006, however, shared care has become increasingly common also among parents with a lower level of education, although it remains quite uncommon if both parents are low educated (Sodermans et al., 2013).

*We hypothesize that the association between education and post-separation distance changes over separation cohorts (H2). We expect that during the 1990s, when shared physical custody was rare, highly educated parents (the most mobile group) lived farthest away from their children. In contrast, from the early 2000s onwards, when shared physical custody became more common, we expect that the geographic distance decreased especially for highly educated parents. Finally, we expect that during the 2010s the geographic distance between parents and children decreased for all educational groups.*

## Care Needs Versus Stronger Bonds: Age of Child and Parent–Child Geographic Proximity

The post-separation reorganization of the family is strongly connected to the age of the child. The effect of child’s age on distance to non-resident parents is, however, not theoretically straightforward. On the one hand, younger children are more demanding in terms of care, and close geographical proximity seems more important so that both parents can substantially contribute to the care. Face-to-face contact between parents and very young children is also more difficult to replace with telecommunication (Viry, 2014). As the children become older and more independently mobile, contact and care might be easier to maintain at greater geographic distances. On the other hand, the parent who moved out while the child was very young had less time to enact the parental role and create a close emotional bond with the child (Cheadle et al., 2010). This might then translate to lower motivation for the parent to stay near and enact the parental role outside of the household. Additionally, mothers are seen as the primary caretakers of the children, especially when they are young. Young children are more likely to stay with their mother, whereas older children have a higher probability of being in shared physical custody or in sole father custody (Sodermans et al., 2013). In terms of distances between children and non-resident parents, similar results have been found. Dommermuth (2016) shows for the Norwegian context that non-resident fathers, and to a somewhat smaller degree also mothers, tend to live farther away when the children are very young (until the age of 4). If the parents separate when the child is older (5–17 for mothers, 7–17 for fathers) parents tend to stay living in closer proximity to their children. There is no research that would show whether the change in proximity that was observed across separation cohorts occurred for children of all ages or whether it is driven by children of a particular age group. However, assuming that geographical proximity is intertwined with shared physical custody trends, *we can hypothesize that the distance between the child and the non-resident parent has become smaller, especially for children who are older at the time of parental separation (H3).*

## Belgian Context

As this research is embedded within the Belgian context, in this section, we provide information about the changes in the parental leave, legal regulations of custody arrangements, and divorce law between 1992 and 2018 as well as information about the basic geography of Belgium and patterns of internal migration.

In terms of parental leave, Belgium introduced in 1997, in response to a European directive, parental leave of up to three months for each parent at a low flat-rate benefit (available only to employees) (Marynissen et al., 2019). In 2009, following a new European directive, leave was extended to four months. The uptake of parental leave among fathers, while remaining relatively low, has been increasing. From 2002 to 2020, the proportion of men taking parental leave increased from 8.3 to 33 percent (Blum et al., 2018; Koslowski et al., 2021).

In terms of legal regulations for custody arrangements, there have been two major changes. In 1995 shared legal custody and in 2006 shared physical custody were established as the default. The new custody law implemented in 2006 stipulates that “equally divided alternating residence should be the initial parenting arrangement considered by the judge when a parent exercising joint parental responsibilities makes such a request, or in circumstances where there is no agreement about arrangements for the children” (Vanassche et al., 2017, p. 550).

Divorce law underwent two major reforms and some minor ones. The first major reform, in 1994, simplified the divorce procedure and made it more accessible for divorcing spouses. The second, in 2007, made the switch to a largely no-fault divorce, with other aspects of divorce, such as maintenance payments, also affected. Minor changes concerning specific aspects of divorce law occurred in 1997, 2000, 2013, 2014, and 2018 (Pintens & Torfs, 2002; Swennen, 2021).

Regarding general mobility patterns, it must be noted that Belgium is a relatively small country (30,528 km^2^), with, from north to south and from west to east, the maximum distance around 200 km. The country is divided into the Dutch-speaking north (Flanders), the French-speaking south (Wallonia), and Brussels, a bilingual enclave within the Flemish region. The linguistic, cultural, and historic differences between Flanders and Wallonia mean that people are reluctant to move across regions (Stillwell et al., 2016). Moving distances are generally short and take place within provinces, given the preference to stay near relatives, social relationships, neighbours, and sports and collective activities situated in the same province (Thomas et al., 2017a, b). Belgium consists of 589^2^ municipalities with an average area of 52 km^2^.

## Data and Methods

Register-based data provide a unique opportunity to follow the whole population of separated parents and the change in their residence following relationship dissolution. This research uses information from Demobel, a dataset provided by Statistics Belgium which combines information from National Population Registers (1992–2020) and the Belgian Census (2001, 2011). Census data provide information, among other areas, on educational attainment. National Population Registers include detailed information on household composition, marital status, and place of residence (municipality) for all Belgian residents as of the first of January of each calendar year. The municipality, the smallest spatial unit in our data, is a meaningful unit of analysis. Municipalities structure people’s everyday lives in important ways. They provide local infrastructure (such as schools and leisure organizations), a sense of identity, and location-specific capital (e.g. friends and relatives living nearby).

### Sample

The analytical sample of children who experienced parental separation between years 1992 and 2018 was constructed as follows. First, we selected all children younger than 17 who were registered at the same address as both of their parents (married or not) at the time *t*. In other words, the child, the mother, and the father must share their household identifier. Second, we define separation as parents not being registered in the same household at *t* + 1 (i.e. not having the same household ID). We only consider cases in which both parents are living in Belgium at *t* + 1, in order to ensure information about their residential situation. To make sure we capture separation rather than other moving behaviour, we exclude cases in which parents were again living together at *t* + 2 (60,743 or 6% of cases). This definition of parental separation is applied for all observed years. Logically, we assumed that the household split is accompanied by a residential move for one or both partners. We do not have information in the data prior to 2000 to be able to test this assumption. From 2000, information about a residential move for either of the parents within the year of separation is available. Thus, from 2000, we applied this additional check to further eliminate noise in the data. Particularly, for 2000 and 2008 we noticed an unusually high number of cases where parents split their household (i.e. had a different household ID at *t* + 1) but did not make a residential move. We decided to exclude such cases from the analysis (N = 27,149 or 2.8% of cases). To determine the residential situation of the children, we look at household composition at *t* + 1 by comparing the household identifier of the child with that of each parent. The child can have either a non-resident father, a non-resident mother, or both.^3^ It is important to note that this distinction refers to the registered reality, meaning that we have no information on actual physical custody arrangements. However, findings from the Netherlands suggest that the registration address is usually the main place of residence (van der Wiel & Kooiman, 2019). We created two samples: one of children with non-resident fathers (*N* = 766,411) and another of children with non-resident mothers (*N* = 163,195). The very small number of children who did not reside with either of the parents is present in both samples.

### Variables

The distance between the child and non-resident parent is measured in two ways. First, we investigate whether the child and parent reside in the same municipality; this is indicated by a binary variable. If the child and parent do not reside in the same municipality, we use a measure of geographical distance between the child’s and the non-resident parent’s municipalities. This distance is measured in km between the municipalities’ town halls.^4^ The limitation of this measure is that the distance between non-resident parent and child is only approximate.^5^ As the variable does not have a normal distribution, we use a log transformation of the variable in the multivariate analysis.^6^ Separation cohort is derived from the year in which parental separation took place and ranges from 1992 to 2018. We introduce separation cohorts as yearly dummies, which is the most flexible specification to study time trends. The educational level of the father and of the mother are derived from censuses with four categories: (1) primary or lower secondary education, (2) higher secondary education, (3) tertiary education, and (4) missing. Census data are only collected in three time points (1991, 2001 and 2011). As the mean age at separation is 36 for mothers and 39 for fathers, it can be assumed that the vast majority of parents had completed their education. Not having the information on educational level in the precise year should thus have little impact on the results. For each separation cohort, we use the educational information from the closest year available.^7^ Child’s age is distributed into four categories: 0–2 years, 3–6 years, 7–12 years, and 13–17 years. We additionally control for age of the father and of the mother, marital status prior *t* separation (0 = cohabitation, 1 = marriage), repartnering of the mother (1 = living with new partner), repartnering of the father (1 = living with new partner), sex of the child, number of siblings (0, 1, 2, 3 +), presence of any half-siblings from the mother’s side (1 = yes) and the father’s side (1 = yes), and region of residence prior to separation (Flanders, Wallonia, Brussels). Apart from the variable on level of education, no other variable contained missing values.

### Analytical Strategy

For the descriptive analysis, we explore the overall trends in the distance between children and their non-resident parents. We look at the proportion of children with a parent living in a different municipality and at the average distance between children and non-resident parents living in different municipalities. Multivariate analysis is applied in order to assess if changes over separation cohorts could be attributed to overall change in migratory behaviour following parental separation or to compositional changes in the group of children with non-resident mothers and fathers. Furthermore, it enables us to observe associations between the key variables (separation cohort, parental education, and age of child) net of other variables. We employ logistic regression models to study the likelihood of a child’s non-resident parent living in the same municipality upon family dissolution and linear regression models to study the distance between municipalities for children whose non-resident parents live in a different municipality. We estimated three different model specifications for these two dependent variables for two samples: children with non-resident fathers and children with non-resident mothers (all together 12 models). The first model includes all control variables, the second adds an interaction variable between level of education and separation cohort to the first model, and the third adds an interaction variable between child’s age and separation cohort to the first model. In addition, we estimated separate models including an interaction between age and separation cohort for children without any siblings and for children with at least one full sibling. We did this additional analysis because the age of children with older and/or younger siblings may bias estimates of the child’s own age. Focusing on single children allows us to better understand the link between child’s own age and distance.

Although we use population data in this research, we referred to inferential statistics in our multivariate analyses. We do so for two reasons: first, we capture snapshots of the Belgian population at particular time points. These snapshots might be considered as a sample of a larger theoretical population. Even though there is no sampling error as in the case of surveys, observed values can still be the result of a chance. Second, by employing inferential statistics we account for the uncertainties connected to modelling itself (e.g. connected to the unobserved variables or the uncertainty of the outcome variable) (Thygesen & Ersbøll, 2014).

Some of the children in the sample are siblings, i.e. have the same set of parents. The dataset is therefore naturally clustered, and we implement clustered standard errors to account for that fact.

## Results

### Descriptive Results

**Fig. 1:**
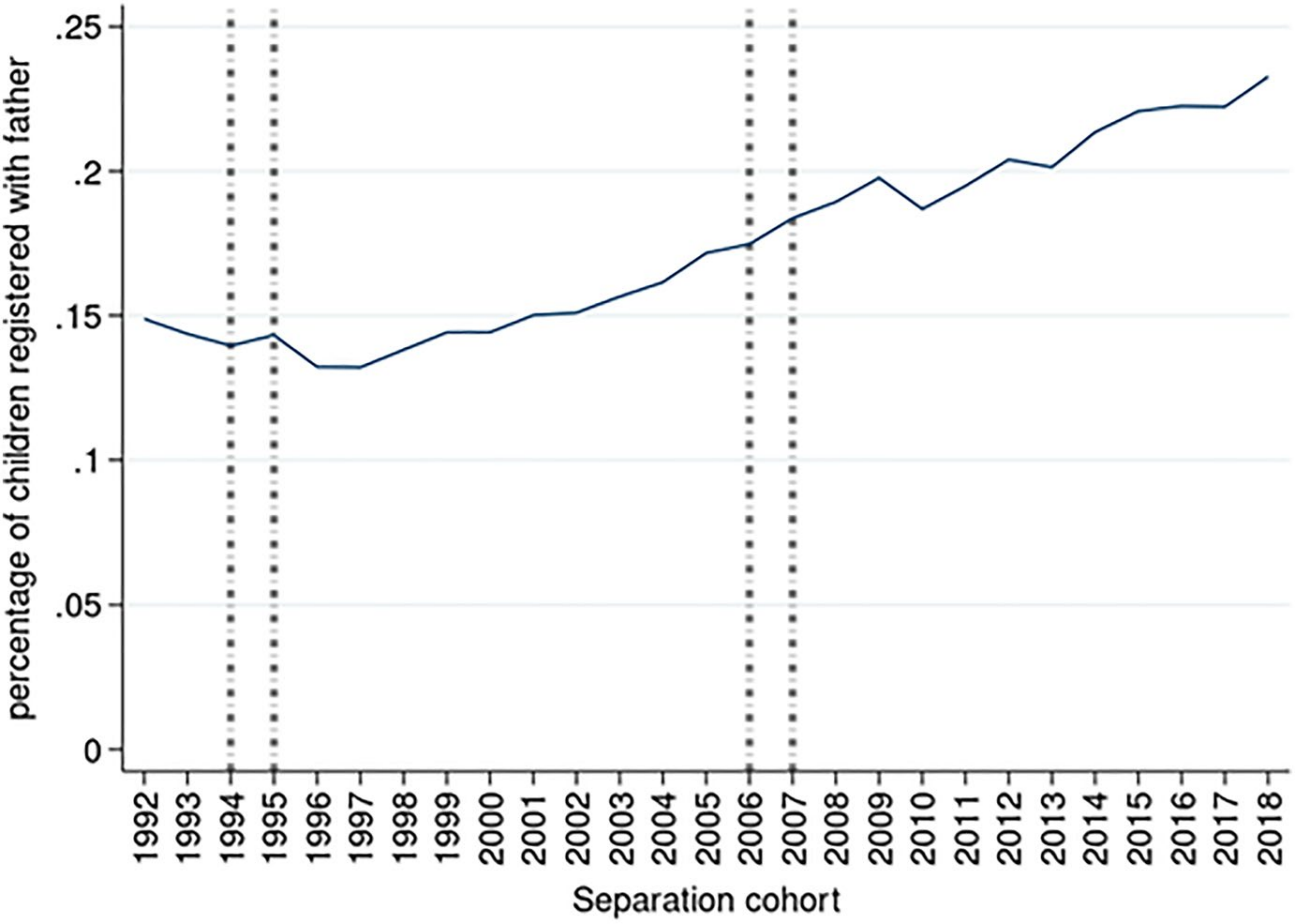
Percentage of children registered with father by separation cohort. *Note*: Vertical lines refer to divorce law reform (1995—shared legal custody, 2006—shared physical custody, 1994 and 2007—major change in divorce law). *Source*: Statbel (Directorate-General Statistics—Statistics Belgium), Demobel (adapted from the National Register), Census 2001, Census 2011, own calculations

Table 1 displays the descriptive statistics of the two samples—children with non-resident fathers and children with non-resident mothers. Older children as compared to younger children more often lived with their fathers. Non-resident parents repartnered in the year immediately after separation (*t* + 1) more often than resident parents, and non-resident mothers repartnered in twice as many cases as non-resident fathers (22% compared to 11%). In the sample of children with non-resident mothers, both parents were older and have tertiary education compared to sample of children with non-resident fathers. The share of non-resident mothers steadily increased from 15% in 1992 to 23% in 2018 (Fig. 1).

**Table 1:** Descriptive statistics

		Non-resident father		Non-resident mother	
		*N*	%	*N*	%
*Categorical variables*					
Level of education—mother	Low	191,560	25	39,826	24
	Medium	256,731	33	51,507	32
	High	220,912	29	53,058	33
	Missing	97,209	13	18,804	12
Level of education—father	Low	231,215	30	42,994	26
	Medium	250,126	33	53,851	33
	High	167,093	22	48,736	30
	Missing	117,978	15	17,614	11
Child’s age	0–2 years	113,033	15	12,122	7
	3–6 years	239,785	31	40,402	25
	7–12 years	263,650	34	59,506	36
	13–17 years	149,944	20	51,165	31
Child’s sex	Male	386,640	50	87,398	54
	Female	379,772	50	75,797	46
Parental union type prior to separation	Cohabitation	203,778	27	41,377	25
	Marriage	562,634	73	121,818	75
Mother lives with a new partner at *t* + 1	No	709,428	93	127,452	78
	Yes	56,984	7	35,743	22
Father lives with a new partner at *t* + 1	No	679,309	89	153,682	94
	Yes	87,103	11	9,513	6
Number of full siblings	0	215,903	28	35,701	22
	1	333,262	43	73,872	45
	2	145,269	19	35,469	22
	3 +	71,978	9	18,153	11
Any half-sibling on mother’s side at *t* + 1		80,227	10	13,780	8
Any half-sibling on father’s side at *t* + 1		80,334	10	14,196	9
Separation cohort	1992	22,612	3	3,960	2
	1993	23,690	3	3,980	2
	1994	25,171	3	4,092	3
	1995	26,707	3	4,466	3
	1996	27,802	4	4,240	3
	1997	28,147	4	4,291	3
	1998	28,025	4	4,497	3
	1999	28,347	4	4,779	3
	2000	28,806	4	4,857	3
	2001	30,839	4	5,449	3
	2002	32,537	4	5,792	4
	2003	33,311	4	6,186	4
	2004	31,644	4	6,103	4
	2005	29,801	4	6,178	4
	2006	28,551	4	6,047	4
	2007	28,334	4	6,379	4
	2008	28,790	4	6,727	4
	2009	29,109	4	7,177	4
	2010	30,499	4	7,011	4
	2011	28,929	4	7,007	4
	2012	27,609	4	7,078	4
	2013	27,696	4	6,985	4
	2014	27,740	4	7,535	5
	2015	27,397	4	7,762	5
	2016	27,685	4	7,928	5
	2017	28,362	4	8,109	5
	2018	28,272	4	8,580	5
Region	Flanders	387,623	51	86,504	53
	Wallonia	305,700	40	65,403	40
	Brussels	73,089	10	11,288	7
Total		766,412	100	163,195	100

	Non-resident father		Non-resident mother	
	Mean(min–max)	Sd	Mean(min–max)	Sd
*Continuous variables*				
Mother’s age	36(16–67)	6.7	37(17–72)	6.5
Father’s age	38(17–87)	7.3	40(16–86)	7.2
Total	766,412		163,195	

*Source*: Statbel (Directorate-General Statistics—Statistics Belgium), Demobel (adapted from the National Register), Census 2001, Census 2011, own calculations

The exploration of the characteristics of children and parents for 1995, 2005, and 2015 (Table 2 in the Appendix) shows that the composition of the children, non-resident fathers, and mothers changed across the cohorts. Both non-resident mothers and fathers had on average higher education in the more recent cohorts. The group of children with non-resident mothers had a higher proportion of children aged 3–6 and 7–12 in the more recent cohorts. The composition of the samples also reflects the increasing popularity of cohabitation as well as the rising mean age at separation of parents.

**Fig. 2:**
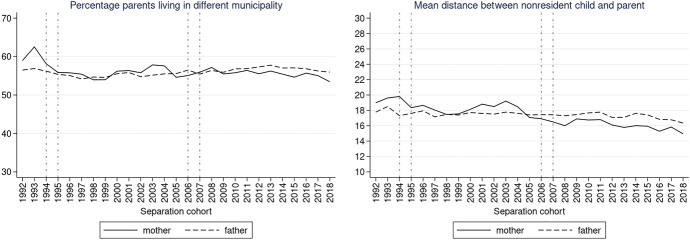
Percentage of children with parents living in a different municipality (left) and mean distance in km between child and parent living in different municipality (right) by separation cohort. *Note*: Vertical lines refer to divorce law reforms (1995—shared legal custody, 2006—shared physical custody, 1994 and 2007—major changes in divorce law). *Source*: Statbel (Directorate-General Statistics—Statistics Belgium), Demobel (adapted from the National Register), Census 2001, Census 2011, own calculations

We further explored the trends in the proportion of children with non-resident fathers and mothers living in a different municipality than the non-resident parent in the year after the separation (Fig. 2, left panel). Throughout the separation cohorts, the proportion of children with non-resident fathers living in a different municipality than the father remained quite stable, between 54 and 58%. Trends were very similar for children with non-resident mothers. The distance between children and non-resident fathers who lived in a different municipality also remained quite stable at around 17.5 km, with a slight decrease of 1.5 km between 2014 and 2018. For children with non-resident mothers, we saw a decrease in distance after 2005, from levels of about 19 km in 1992 to 15 km in 2018.

To explore the trends according to our variables of interest (parental education and child’s age), we also plotted the same graphs for different educational groups of non-resident parents as well as for the different age groups of children. The description of the results and the respective Figs. (9, 10, 11, 12, 13, 14) are presented in the Appendix.

## Multivariate Results

**Fig. 3:**
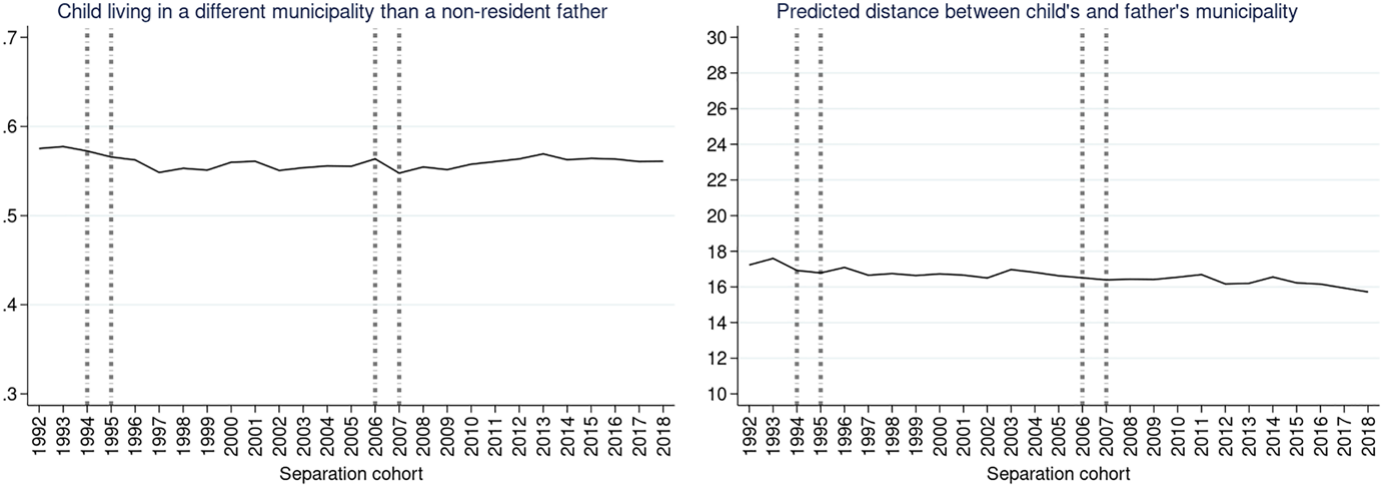
Predicted probabilities of a child living in a different municipality than a non-resident father (left) and predicted distance between the different municipalities in km (right). *Note*: Calculated from model M1 in Table 2 in the Appendix. Vertical lines refer to divorce law reforms. *Source*: Statbel (Directorate-General Statistics—Statistics Belgium), Demobel (adapted from the National Register), Census 2001, Census 2011, own calculations

**Fig. 4:**
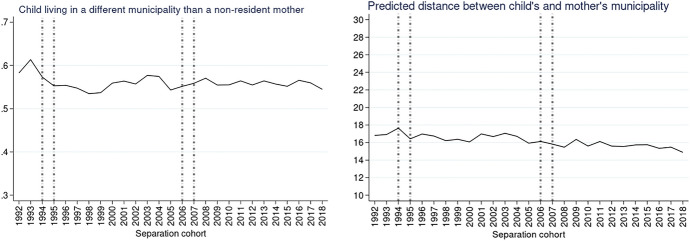
Predicted probabilities of a child living in a different municipality than a non-resident mother (left) and predicted distance between the different municipalities in km (right). *Note*: Calculated from model M1 in Table 2 in Appendix. Vertical lines refer to divorce law reforms. *Source*: Statbel (Directorate-General Statistics—Statistics Belgium), Demobel (adapted from the National Register), Census 2001, Census 2011, own calculations

**Fig. 5:**
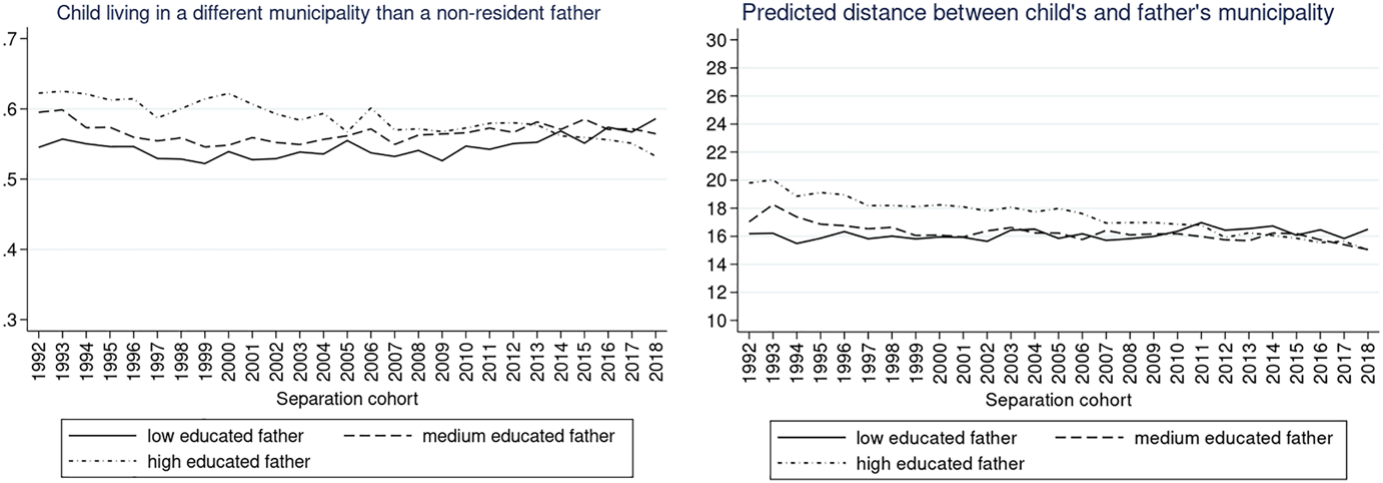
Predicted probabilities of a child living in a different municipality than a non-resident father (left) and predicted distance between the different municipalities in km (right) by father’s level of education. *Source*: Statbel (Directorate-General Statistics—Statistics Belgium), Demobel (adapted from the National Register), Census 2001, Census 2011, own calculations

**Fig. 6:**
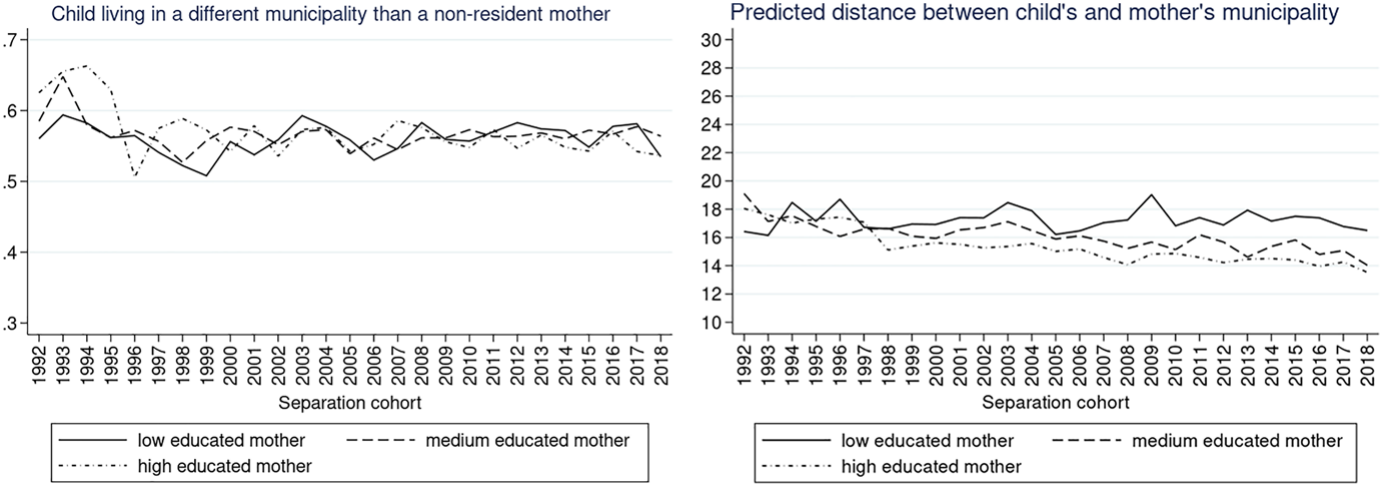
Predicted probabilities of a child living in a different municipality than a non-resident mother (left) and predicted distance between the different municipalities in km (right) by mother’s level of education. *Source*: Statbel (Directorate-General Statistics—Statistics Belgium), Demobel (adapted from the National Register), Census 2001, Census 2011, own calculations

**Fig. 7:**
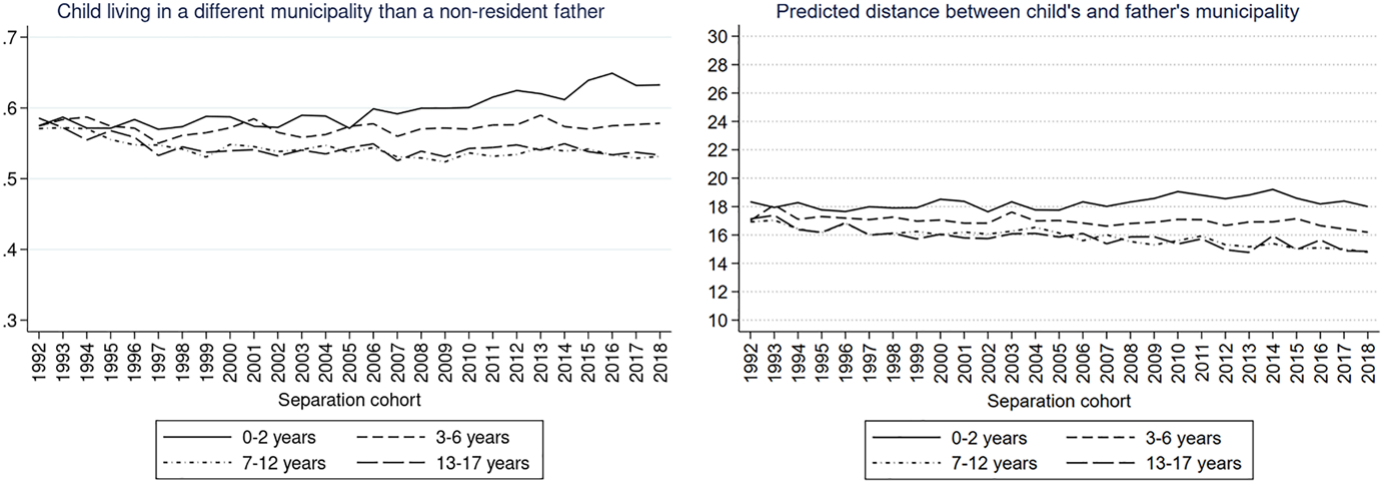
Predicted probabilities of a child living in a different municipality than a non-resident father (left) and predicted distance between the different municipalities in km (right) by child’s age. *Source*: Statbel (Directorate-General Statistics—Statistics Belgium), Demobel (adapted from the National Register), Census 2001, Census 2011, own calculations

**Fig. 8:**
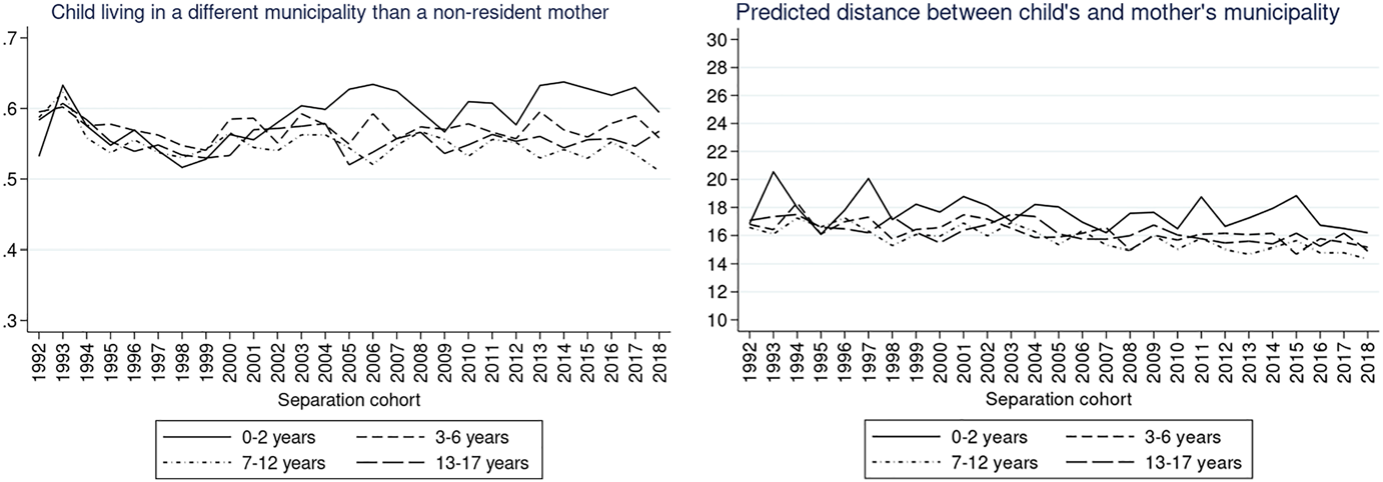
Predicted probabilities of a child living in a different municipality than a non-resident mother (left) and predicted distance between the different municipalities in km (right) by child’s age. *Source*: Statbel (Directorate-General Statistics—Statistics Belgium), Demobel (adapted from the National Register), Census 2001, Census 2011, own calculations

**Fig. 9:**
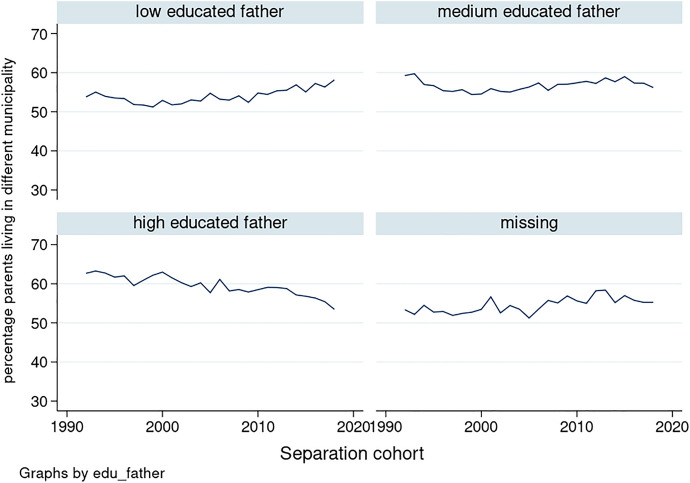
Percentage of children with fathers living in a different municipality by fathers’ level of education and separation cohort. *Source*: Statbel (Directorate-General Statistics—Statistics Belgium), Demobel (adapted from the National Register), Census 2001, Census 2011, own calculations

**Fig. 10:**
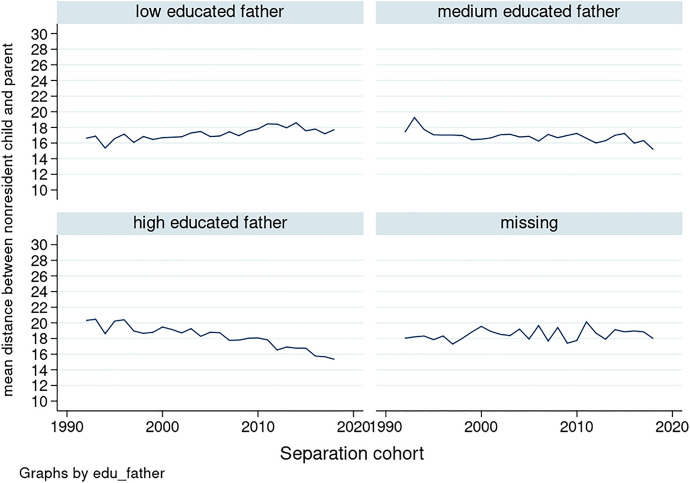
Mean distance in km between children and fathers living in a different municipality by fathers’ level of education and separation cohort. *Source*: Statbel (Directorate-General Statistics—Statistics Belgium), Demobel (adapted from the National Register), Census 2001, Census 2011, own calculations

**Fig. 11:**
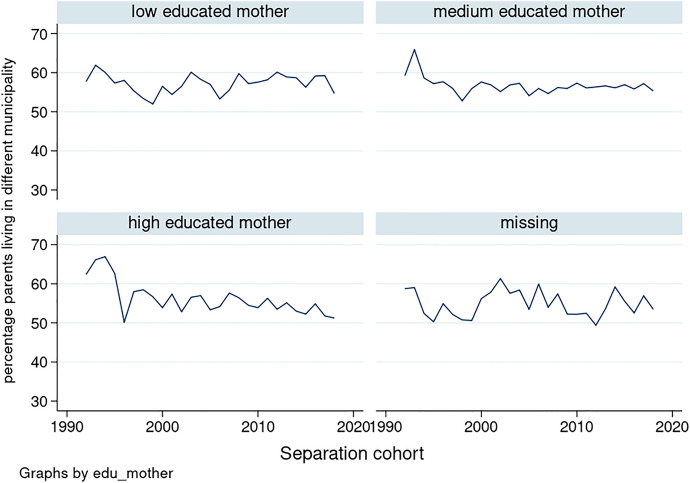
Percentage of children with mothers living in a different municipality by mothers’ level of education and separation cohort. *Source*: Statbel (Directorate-General Statistics—Statistics Belgium), Demobel (adapted from the National Register), Census 2001, Census 2011, own calculations

**Fig. 12:**
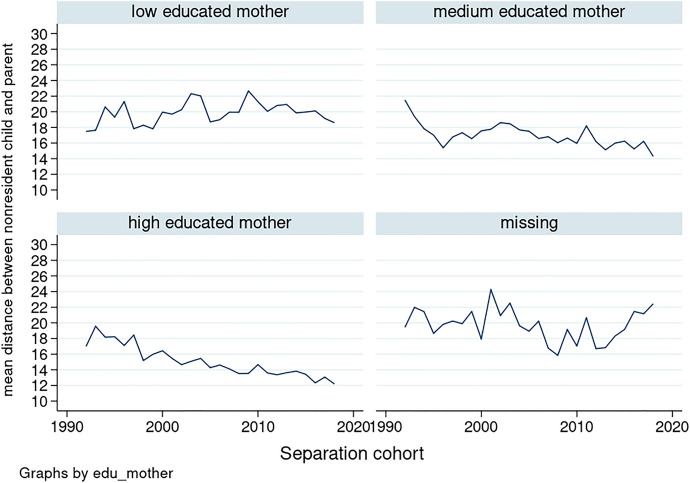
Mean distance in km between children and mothers living in a different municipality by mothers’ level of education and separation cohort. *Source*: Statbel (Directorate-General Statistics—Statistics Belgium), Demobel (adapted from the National Register), Census 2001, Census 2011, own calculations

**Fig. 13:**
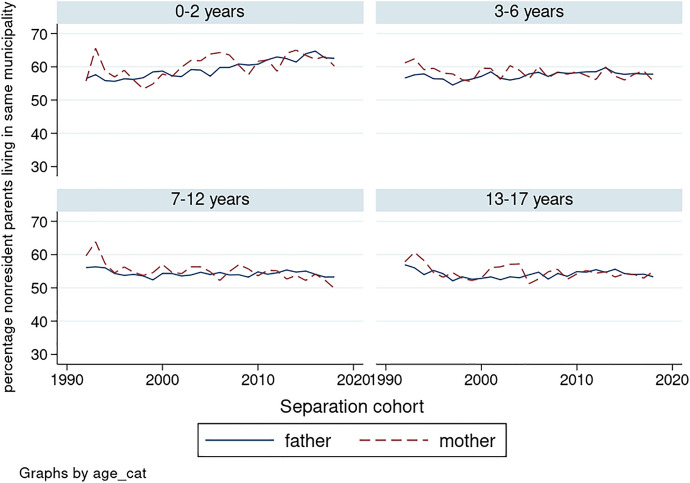
Percentage of children with parents living in a different municipality by children’s age and separation cohort. *Source*: Statbel (Directorate-General Statistics—Statistics Belgium), Demobel (adapted from the National Register), Census 2001, Census 2011, own calculations

**Fig. 14:**
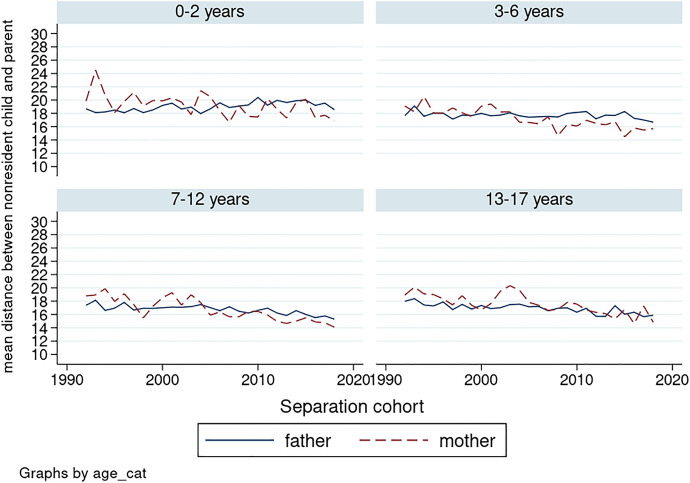
Mean distance in km between children and parents living in a different municipality by children’s age and separation cohort. *Source*: Statbel (Directorate-General Statistics—Statistics Belgium), Demobel (adapted from the National Register), Census 2001, Census 2011, own calculations

In order to explore the change in distances across separation cohorts and the associations with parental education as well as child’s age net of other characteristics, we present the results of logistic regressions (Y = living in a different municipality) and linear regressions (Y = log-transformed distance between municipalities). Figures 3, 4, 5, 6, 7, 8 present predicted probabilities at specified values of covariates of interest (i.e. separation cohort, education, and age of child) and summed to a weighted average reflecting the distribution of the confounders in the target population. We present the models without interaction terms in the Appendix (Tables 3 and 4).

### Trends Over Separation Cohorts

Similar to the descriptive findings, the multivariate estimates of the predictive margins suggest that there was relatively little change across separation cohorts in the probability that the child and non-resident father live in a different municipality (Fig. 3, left panel): predicted probabilities oscillate between 0.54 and 0.58. We observe that among children with a father living in a different municipality, fathers tend to live in somewhat closer proximity in more recent separation cohorts; however, this change is very small (1.2 km difference in predicted mean distance between 1992 and 2018; Fig. 3, right panel).

The predicted probability that a non-resident mother lived in a different municipality than the child peaked in 1992 at 0.59 and then stabilized at around 0.56, eventually reaching a minimum of 0.53 in 2018 (Fig. 4). The predicted distance between children and non-resident mothers living in a different municipality than their child decreased from 17 km in 1992 to 15 km in 2018. While mothers lived on average farther from their children than fathers at the beginning of the observation period. Towards the end of the observation period, mothers lived in closer proximity to their children than fathers.

In the case of non-resident fathers as well as non-resident mothers, the multivariate findings are similar to the descriptive findings, meaning that the trend is not driven by compositional changes in the group of children and non-resident fathers or mothers. The observed decrease in distance, while small, is in line with our hypothesis (H1).

### Trends Over Separation Cohorts by Parental Educational Attainment

We predicted trends in the likelihood that children live in the same municipality as their non-resident father for different levels of their father’s education (Fig. 5, left). Compared to other children, children with a highly educated father were most likely to live in a different municipality than their father in the 1990s and least likely at the end of the 2020s (predicted probability 0.62 in 1992 and 0.54 in 2018). A similar contrast between children with low- and high-educated fathers can be seen in the distance the father lives away if living in a different municipality (Fig. 5, right). While there is a clear decrease in distance from 20 to 15 km for children with highly educated fathers, for children with a low-educated father, the distance oscillated throughout the whole observation window between 15 and 17 km.

Unlike in the case of non-resident fathers, the predicted probabilities of a child living in a different municipality than their mother do not follow distinctly different trends for mothers with different levels of education (Fig. 6, left). However, for non-resident mothers living in a different municipality, the predicted mean distance decreased foremost for highly educated women (from 18 km in 1992 to 14 km in 2018) and medium-educated women (from 19 km in 1992 to 14 km in 2018), but not for low-educated mothers (16 km in 1992 and 2018).

These results are in line with our hypothesis that the association between education and post-separation distance varies over separation cohorts (H2) as well as with our expectations that the distance would decrease foremost for children with highly educated parents since the 2000s. Nevertheless, contrary to expectations, there is no evidence that the distance was decreasing for children with low-educated parents: considering the increasing proportion of children with low-educated fathers living in a different municipality, the distance is in fact increasing, especially in the 2010–2018 period.

### Trends Over Separation Cohorts by Child’s Age

When estimating the trends in distance between children and their non-resident fathers for children of different age groups (Fig. 7), we see contrasting developments. While at the beginning of the 1990s, children of all ages had a similar probability of living in a different municipality than their father, we observe stark differences across the age groups throughout the 2010s (Fig. 7, left). For the youngest children, aged 0–2, the probability of living in a different municipality than the father increased from 0.56 in 1992 to 0.62 in 2018. For the children aged 3–6 years, the probability remained roughly the same throughout the observed period, while for the older children the probability decreased from about 0.57 in 1992 to 0.53 in 2018. Among children with a father living in a different municipality, the distance to the father decreased, even though only modestly, except if the child was very young (Fig. 7, right).

**Fig. 15:**
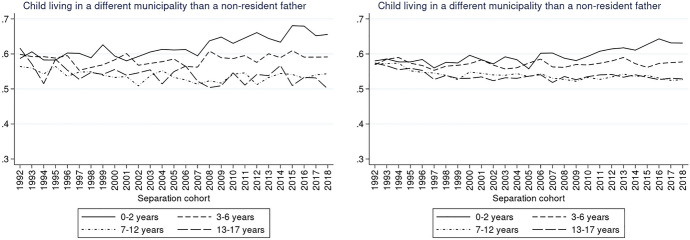
Predicted probabilities of a child living in a different municipality than a non-resident father if the child is the only child (left) and predicted probabilities of a child living in a different municipality than a non-resident father if the child has any siblings (right), by child’s age. *Source*: Statbel (Directorate-General Statistics—Statistics Belgium), Demobel (adapted from the National Register), Census 2001, Census 2011, own calculations

The emerging differences according to the child’s age might be difficult to understand considering that some of the children have older or younger siblings and the distance between the child and non-resident parent might be also influenced by the age of the sibling. To better understand the effect of age, we ran separate models predicting the probability of living in different municipality including only children without siblings (Appendix, Fig. 15, left) and at least one full sibling (Appendix, Fig. 15, right). In general, we see that the observed general pattern is reflected in both groups of children. In recent separation cohorts, children aged 0–2 years were more likely to have a father living in a different municipality than older children, regardless of the presence of siblings. Nevertheless, children aged 0–2 without siblings were especially likely to live in a different municipality than the father.

Figure 8 shows the trends in distance for children with non-resident mothers of different age groups. A gap between the age groups in the probability of living in a different municipality can be discerned from the beginning of the 2000s (Fig. 8, left). In these more recent separation cohorts, compared to children older than 3 years, very young children (0–2 years) were more likely to have a mother living in a different municipality. In the same vein, if the mother lived in a different municipality she was slightly more likely to live farther away if the child was 0–2 compared to older ages. This difference remained relatively stable over separation cohorts (Fig. 8, right).

**Fig. 16:**
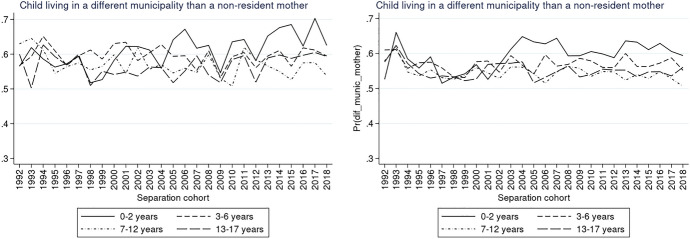
Predicted probabilities of a child living in a different municipality than a non-resident mother if the child is the only child (left) and predicted probabilities of a child living in a different municipality than a non-resident mother if the child has any siblings (right), by child’s age. *Source*: Statbel (Directorate-General Statistics—Statistics Belgium), Demobel (adapted from the National Register), Census 2001, Census 2011, own calculations

Separate models predicting the probability of living in a different municipality than a non-resident mother for children without any siblings and for those with at least one full sibling show less conclusive results compared to the case of non-resident fathers (Appendix, Fig. 16). Children aged 0–2 were more likely to live farther from their non-resident mother than older children, especially in more recent cohorts, regardless of the presence of siblings. Nevertheless, the estimates show large fluctuations across separation cohorts, which can be attributed to the relatively small number of cases of children with non-resident mothers, especially if they are quite young.

The results of children with non-resident fathers and mothers are generally in line with our hypothesis H3 that the distances decreased particularly for older children.

### Other Notable Findings

While our research focused on the trends in distances between children and their non-resident parents over separation cohorts with attention to parental educational attainment and child’s age, here we comment on other notable findings which stood out from our analysis. Parental repartnering is associated strongly with greater distances between children and their non-resident parents. This association is present for both non-resident fathers and mothers and regardless of which parent repartners (i.e. the resident or non-resident parent). Further, we also observed a negative association between presence of any half-sibling (from the side of mother of father) and distance to a non-resident parent. These findings could suggest that family complexity makes it more difficult to maintain close proximity between children and their non-resident parent.

## Discussion

This paper explored the trends in the distance between children and their non-resident parent following the residential separation of their parents. We analysed Belgian register–based data including separation cohorts from 1992 to 2018. Changing patterns of care distribution between parents within this period were hypothesized as having important implications for the location choice of parents following the separation. We explored the overall trends for non-resident mothers and fathers as well as for different parental educational groups and parents with children of different ages.

Our results showed relatively little change in the overall distance between non-resident parents and children, even though this period was marked by profound changes in post-divorce families. We observed only a small decrease in the distance between children and their non-resident father while a somewhat larger decrease for non-resident mothers. The decrease in the distance for non-resident mothers seems to be linked to the increase in the overall number of non-resident mothers, presumably driven by the expansion of shared physical custody arrangements. Although we do not test the effect of policies on the post-separation geographical distances, from the visual examination of the trends in distance over the separation cohorts were mostly gradual rather than shock effects following policy interventions. Nevertheless, the potential effect of the policies is yet to be established.

The modest changes in the trends in geographical distance between children and their non-resident parents were in line with the results of the Swedish study following the 1990s (Turunen et al., 2023). The fact that we find essentially the same result as in the Swedish context is remarkable, as Sweden and Belgium differ in number of aspects, including geographies, mobility patterns (shorter distance moves more prevalent in Belgium (Stillwell et al., 2016)), childcare patterns (Belgian father being much less likely to use father’s leave (Marynissen et al., 2019)) and prevalence of shared custody (in Belgium less common (Steinbach et al., 2021)) as well as timing of the changes (Sweden being often labelled as a forerunner of decreasing gender inequality and increasing men’s involvement (Evertsson, 2014)).

Although we observed only modest changes over separation cohorts in the overall distance between children and their non-resident parents, we noted important changes across separation cohorts for non-resident parents of different educational groups as well as for children of different ages. For older children and children with higher-educated non-resident parents, the distance to the non-resident parent decreased substantially in more recent separation cohorts. These findings are in line with trends in shared physical custody arrangements whereby shared custody has become more common among higher-educated parents and among parents with somewhat older children (Sodermans et al., 2013). For parents with very young children (0–2 years) or low-educated fathers, the distance slightly increased between 1992 and 2018. These findings point to inequalities of certain parent–child pairs and have important implications for children as well as parents.

We observed that the distance to the father has slightly increased for children with lower-educated fathers. We can speculate that this could be the result of two different factors. First, low-educated fathers may have fewer resources than higher-educated fathers, making it more difficult to live nearby their children (e.g. finding appropriate housing in the proximity of the children could be increasingly difficult). Second, low-educated fathers may be less involved in childrearing and be less motivated to live near to their children compared to more educated fathers, especially in more recent years. As our research is unable to identify the underlying cause of the contrasting trends according to paternal education, this would require further investigation. Nevertheless, as children of low-educated parents are more likely to experience parental separation (Kalmijn & Leopold, 2021) and low-educated non-resident fathers are increasingly more likely to live farther away, it points to the increasing disadvantage of children of low-educated parents.

As for the children who experience parental separation at a very young age, our findings point to a concerning trend. As children aged 0–2 years are increasingly living farther away from their non-resident parent, parental separation seems to increasingly limit the availability of the non-resident parent throughout a child’s life course. The ties between the non-resident parent and potentially other members of the non-resident parent’s family and child may deteriorate to a greater extent in more recent separation cohorts. Young children may be thus increasingly losing support and contact with their non-resident parent and that parent’s extended family.

The present study has some limitations. Although register data provide information on the whole population and are an invaluable source of information, they refer to the registered reality, which might not always correspond to the actual situation. Furthermore, the data do not provide an exact measure of the distance between the household of the non-resident parent and the child, and distance could only be measured at the municipality level. We were also lacking data on custody arrangements, and some of the parents labelled as non-resident are parents with shared physical custody. Finally, we were limited to measuring education at only three time points. Despite these limitations, our study makes an important contribution to understanding the geographical reorganization of post-separation families in Belgium and how this has evolved over 27 years. It pointed out that even though for some child–non-resident-parent pairs the distance decreased, for some it increased. The change in geographic distance has complex implications for instrumental support, frequency, and quality of non-resident-parent–child contact.

## Data Availability

We used secondary data for our research called DEMOBEL data (adapted from the Belgian National register) for this research. The datasets analysed during the current study are not publicly available. The data are not publicly available and subject to approval by the data provider Statbel (Belgian statistical office). Upon request, we can share details about datasets and variables used in this analysis which can be requested from Statbel. Code used for the analysis of the data is available also on request.

## Appendix

See Table

**Table 2:** Descriptive statistics for selected separation cohorts

		Non-resident fathers			Non-resident mothers		
		1995	2005	2015	1995	2005	2015
		%	%	%	%	%	%
*Categorical variables*							
Level of education—mother	Low	26	25	21	29	27	18
	Medium	31	37	30	26	35	31
	High	16	28	38	14	29	44
	Missing	28	10	11	31	9	7
Level of education—father	Low	31	30	28	31	28	20
	Medium	28	36	33	28	33	34
	High	14	23	26	16	29	39
	Missing	27	12	13	24	10	7
Child’s age	0–2 years	15	14	15	8	7	7
	3–6 years	32	30	33	22	23	27
	7–12 years	34	35	34	34	36	38
	13–17 years	19	22	19	36	34	27
Child’s sex	Male	51	50	51	55	54	53
	Female	49	50	49	45	46	47
Parental union type prior to separation	Cohabitation	13	24	43	10	19	38
	Marriage	87	76	57	90	81	62
Mother lives with a new partner	No	93	93	92	76	77	80
	Yes	7	7	8	24	23	20
Father lives with a new partner	No	90	89	88	93	94	95
	Yes	10	11	12	7	6	5
Number of full siblings	0	29	28	29	24	21	21
	1	43	43	44	41	44	48
	2	18	19	19	21	21	22
	3 +	10	10	9	13	14	9
Any half-sibling, mother’s side		8	10	13	8	8	9
Any half-sibling, father’s side		8	10	14	9	8	10
Region	Flanders	50	51	51	50	52	53
	Wallonia	41	40	39	41	41	41
	Brussels	9	9	10	9	6	6

		mean	mean	mean	mean	mean	mean
*Continuous variables*							
Mother’s age		34	36	37	35	37	38
Father’s age		36	39	40	39	41	41
Observations		26,707	29,801	27,397	4,466	6,178	7,762

*Source*: Statbel (Directorate-General Statistics—Statistics Belgium), Demobel (adapted from the National Register), Census 2001, Census 2011, own calculations

2

## Descriptive Analysis

Figure 9 shows that the proportion of fathers living in a different municipality than the child was slightly increasing for the low-educated fathers from the early 2000s, but steadily decreasing for highly educated fathers (from about 63% in 1992 to 54% in 2018). Highly educated fathers were the group most likely to live in a different municipality during the 1990s but the lowest in the most recent cohorts. Very similar trends could be observed for the distance between a father and a child (Fig. 10). The trends for the different educational levels of non-resident mothers (Fig. 11) are less clear, with more fluctuations observed between the individual years. The proportion of those living in a different municipality was similar across different educational levels of mothers. On the other hand, the distance between a mother and a child (Fig. 12) decreased foremost for highly educated mothers from around 20 km in 1993 to 12 km in 2018. For comparison, the distance for medium-educated mothers decreased from 22 km (1992) to 14 km (2018), and for low-educated mothers oscillated between 17 and 22 km. When displaying the trends by child’s age (Figs. 13 and 14), we observe that the fathers and mothers of the youngest children (0–2 years) increasingly resided in different municipality than their child (Fig. 13), while for other age groups we see little change. When looking at distances (Fig. 14), fathers, but not mothers, of children aged 0–2 lived increasingly farther away, while the trend had a slightly decreasing tendency for the older children.

See Tables

**Table 3:** Logistic regressions with a binary dependent variable indicating if a parent lives in a different municipality than their child

		Non-resident fathers		Non-resident mothers	
		OR	CI	OR	CI
Level of education—mother	Low (ref)				
	Medium	1.14***	(1.12–1.16)	1.03	(0.99–1.07)
	High	1.24***	(1.21–1.26)	1.01	(0.96–1.05)
	Missing	0.96***	(0.94–0.99)	0.90***	(0.86–0.95)
Level of education—father	Low (ref)				
	Medium	1.09***	(1.07–1.11)	1.02	(0.99–1.06)
	High	1.17***	(1.15–1.20)	1.02	(0.98–1.07)
	Missing	1.00	(0.98–1.03)	1.01	(0.95–1.06)
Child’s age	0–2 years (ref)				
	3–6 years	0.90***	(0.89–0.91)	0.91***	(0.87–0.95)
	7–12 years	0.80***	(0.78–0.81)	0.82***	(0.78–0.86)
	13–17 years	0.80***	(0.79–0.82)	0.85***	(0.80–0.90)
Child’s sex	Male (ref)				
	Female	1.01***	(1.00–1.02)	1.01	(0.99–1.03)
Number of full siblings	0 (ref)				
	1	0.94***	(0.93–0.95)	0.92***	(0.89–0.95)
	2	0.92***	(0.91–0.94)	0.91***	(0.87–0.94)
	3 +	0.94***	(0.91–0.97)	1.00	(0.94–1.06)
Any half-sibling, father’s side		1.06***	(1.04–1.08)	1.07***	(1.02–1.12)
Any half-sibling, mother’s side		1.07***	(1.05–1.09)	1.06***	(1.02–1.11)
Mother’s age		1.01***	(1.01–1.01)	1.00**	(0.99–1.00)
Father’s age		0.99***	(0.99–0.99)	1.00**	(0.99–1.00)
Parental union type prior to separation	Cohabitation (ref)				
	Marriage	1.04***	(1.02–1.06)	1.06***	(1.03–1.10)
Mother lives with a new partner	No (ref)				
	Yes	1.47***	(1.43–1.51)	1.76***	(1.70–1.83)
Father lives with a new partner	No (ref)				
	Yes	1.72***	(1.68–1.76)	1.26***	(1.19–1.34)
Separation cohort	1992 (ref)				
	1993	1.01	(0.96–1.06)	1.14**	(1.01–1.29)
	1994	0.99	(0.94–1.04)	0.96	(0.85–1.09)
	1995	0.96	(0.91–1.01)	0.88**	(0.78–1.00)
	1996	0.95**	(0.90–1.00)	0.89*	(0.79–1.00)
	1997	0.89***	(0.85–0.94)	0.86**	(0.77–0.98)
	1998	0.91***	(0.87–0.96)	0.82***	(0.73–0.93)
	1999	0.90***	(0.86–0.95)	0.83***	(0.73–0.93)
	2000	0.94**	(0.89–0.99)	0.91	(0.80–1.02)
	2001	0.94**	(0.90–0.99)	0.92	(0.82–1.04)
	2002	0.90***	(0.86–0.95)	0.90*	(0.80–1.01)
	2003	0.91***	(0.87–0.96)	0.98	(0.87–1.09)
	2004	0.92***	(0.88–0.97)	0.97	(0.86–1.08)
	2005	0.92***	(0.88–0.97)	0.85***	(0.76–0.95)
	2006	0.95*	(0.91–1.00)	0.88**	(0.78–0.99)
	2007	0.89***	(0.85–0.94)	0.91*	(0.81–1.01)
	2008	0.92***	(0.87–0.97)	0.95	(0.85–1.06)
	2009	0.91***	(0.86–0.95)	0.89**	(0.80–0.99)
	2010	0.93***	(0.88–0.98)	0.89**	(0.80–1.00)
	2011	0.94**	(0.89–0.99)	0.93	(0.83–1.04)
	2012	0.95*	(0.91–1.00)	0.89**	(0.80–1.00)
	2013	0.98	(0.93–1.03)	0.93	(0.83–1.04)
	2014	0.95**	(0.90–1.00)	0.90*	(0.80–1.00)
	2015	0.96*	(0.91–1.01)	0.88**	(0.79–0.98)
	2016	0.95*	(0.90–1.00)	0.93	(0.84–1.04)
	2017	0.94**	(0.89–0.99)	0.91*	(0.81–1.01)
	2018	0.94**	(0.90–0.99)	0.85***	(0.77–0.95)
Region	Flanders				
	Wallonia	1.18***	(1.16–1.19)	1.28***	(1.25–1.32)
	Brussels	1.63***	(1.59–1.67)	1.61***	(1.52–1.71)
Constant		1.14***	(1.08–1.21)	1.61***	(1.42–1.83)
Observations		766,412		163,195	

^*^*p* < .05; ***p* < .01; ****p* < .001

^3^ and

**Table 4:** Linear regression fitting the logged distance between municipalities of parent and child

		Non-resident fathers		Non-resident mothers	
		Coeff	CI	Coeff	CI
Level of education—mother	Low (ref)				
	Medium	− 0.02***	(− 0.03 to − 0.01)	− 0.07***	(− 0.09 to − 0.05)
	High	0.01	(− 0.00–0.02)	− 0.13***	(− 0.15 to − 0.11)
	Missing	0.01	(− 0.00–0.02)	0.01	(− 0.02–0.04)
Level of education—father	Low (ref)				
	Medium	0.01	(− 0.00–0.01)	− 0.01	(− 0.03–0.01)
	High	0.06***	(0.05–0.07)	− 0.02*	(− 0.04–0.00)
	Missing	0.06***	(0.05–0.07)	0.03*	(-0.00–0.06)
Child’s age	0–2 years (ref)				
	3–6 years	− 0.07***	(− 0.0 to − 0.06)	− 0.08***	(− 0.10 to − 0.06)
	7–12 years	− 0.13***	(− 0.14 to − 0.12)	− 0.11***	(− 0.13 to − 0.08)
	13–17 years	− 0.13***	(− 0.14 to − 0.12)	− 0.08***	(− 0.10 to − 0.05)
Child’s sex	Male (ref)				
	Female	0	(− 0.00–0.01)	0.01	(-0.00–0.02)
Number of full siblings	0 (ref)				
	1	− 0.02***	(− 0.02 to − 0.01)	− 0.03***	(− 0.04 to − 0.01)
	2	0.02***	(0.01–0.03)	0.01	(− 0.02–0.03)
	3 +	0.08***	(0.07–0.10)	0.09***	(0.06–0.13)
Any half-sibling, father’s side	0.06***		(0.05–0.07)	0.08***	(0.06–0.10)
Any half-sibling, mother’s side	0.07***		(0.06–0.08)	0.08***	(0.06–0.11)
Mother’s age		0	(− 0.00–0.00)	− 0.00***	(− 0.01–-0.00)
Father’s age		0.00***	(0.00–0.00)	0.00***	(0.00–0.00)
Parental union type prior to separation	Cohabitation (ref)				
	Marriage	− 0.01	(− 0.01–0.00)	− 0.02*	(− 0.03–0.00)
Mother lives with a new partner	No (ref)				
	Yes	0.11***	(0.10–0.12)	0.17***	(0.15–0.19)
Father lives with a new partner	No (ref)				
	Yes	0.17***	(0.16–0.18)	0.11***	(0.08–0.14)
Separation cohort	1992 (ref)				
	1993	0.02	(− 0.01–0.05)	0.01	(− 0.06–0.07)
	1994	− 0.02	(− 0.04–0.01)	0.04	(− 0.02–0.11)
	1995	− 0.02*	(− 0.05–0.00)	− 0.02	(− 0.08–0.04)
	1996	− 0.01	(− 0.03–0.02)	0.01	(− 0.06–0.07)
	1997	− 0.03**	(− 0.06 to − 0.00)	0	(− 0.07–0.06)
	1998	− 0.03*	(− 0.05–0.00)	− 0.03	(− 0.09–0.03)
	1999	− 0.03**	(− 0.06 to − 0.00)	− 0.02	(− 0.09–0.04)
	2000	− 0.03*	(− 0.05–0.00)	− 0.04	(− 0.10–0.02)
	2001	− 0.03**	(− 0.05 to − 0.00)	0.01	(− 0.05–0.07)
	2002	− 0.04***	(− 0.06 to − 0.01)	− 0.01	(− 0.07–0.05)
	2003	− 0.01	(− 0.04–0.01)	0.02	(− 0.04–0.08)
	2004	− 0.02	(− 0.05–0.00)	0	(− 0.06–0.06)
	2005	− 0.03**	(− 0.06 to − 0.01)	− 0.05	(− 0.11–0.01)
	2006	− 0.04***	(− 0.06 to − 0.01)	− 0.04	(− 0.10–0.02)
	2007	− 0.04***	(− 0.07 to − 0.02)	− 0.06*	(− 0.11–0.00)
	2008	− 0.04***	(− 0.07 to − 0.02)	− 0.08***	(− 0.13 to − 0.02)
	2009	− 0.04***	(− 0.07 to − 0.02)	− 0.02	(− 0.08–0.03)
	2010	− 0.04***	(− 0.06 to − 0.01)	− 0.07**	(− 0.12 to − 0.01)
	2011	− 0.03**	(− 0.05 to − 0.00)	− 0.04	(− 0.10–0.02)
	2012	− 0.06***	(− 0.08 to − 0.03)	− 0.07**	(− 0.13 to − 0.01)
	2013	− 0.06***	(− 0.08 to − 0.03)	− 0.07**	(− 0.13 to − 0.02)
	2014	− 0.04***	(− 0.06 to − 0.01)	− 0.06**	(− 0.12 to − 0.01)
	2015	− 0.05***	(− 0.08 to − 0.03)	− 0.06**	(− 0.12 to − 0.00)
	2016	− 0.06***	(− 0.08 to − 0.03)	− 0.08***	(− 0.14 to − 0.03)
	2017	− 0.07***	(− 0.10 to − 0.05)	− 0.08***	(− 0.13 to − 0.02)
	2018	− 0.09***	(− 0.11 to − 0.06)	− 0.11***	(− 0.16 to − 0.05)
Region	Flanders				
	Wallonia	0.04***	(0.04–0.05)	0.09***	(0.08–0.10)
	Brussels	− 0.67***	(− 0.69 to − 0.66)	− 0.55***	(− 0.59 to − 0.52)
Constant		2.57***	(2.55–2.60)	2.68***	(2.61–2.75)
Observations		428,936		91,271	

**p* < .05; ***p* < .01; ****p* < .001

^4^.

See Figs. 15 and 16

## Footnotes

1 Other components of socio-economic status, such as occupation and income, are correlated with educational attainment, but can have the influence on the geographic proximity on its own.

2 589 municipalities between 1992 and 2018, 581 municipalities from 2019.

3 In a very small number of cases (N = 69) the child resided with neither the mother nor the father following parental separation.

4 In 2019, 15 municipalities merged into 7 new municipalities. As the new municipalities were prone to keep one of the existing town halls, we track the location of the new town hall and retain the information on distances from previous years.

5 The data do not entail an information about the exact location of the dwellings.

6 In one case where the distance between the town halls was 0, the value was imputed with 1.

7 Educational level from the 1991 census is used for separation cohorts 1992–1996, 2001 census is used for separation cohorts 1997–2006, and from the 2011 census for separation cohorts 2007–2018.
